# Analysis of Cannabis Seizures in NSW, Australia: Cannabis Potency and Cannabinoid Profile

**DOI:** 10.1371/journal.pone.0070052

**Published:** 2013-07-24

**Authors:** Wendy Swift, Alex Wong, Kong M. Li, Jonathon C. Arnold, Iain S. McGregor

**Affiliations:** 1 National Drug and Alcohol Research Centre, University of New South Wales, Sydney, NSW, Australia; 2 Discipline of Pharmacology, University of Sydney, Sydney, NSW, Australia; 3 Brain and Mind Research Institute, University of Sydney, Sydney, NSW, Australia; 4 School of Psychology, University of Sydney, Sydney, NSW, Australia; The Scripps Research Institute, United States of America

## Abstract

Recent analysis of the cannabinoid content of cannabis plants suggests a shift towards use of high potency plant material with high levels of Δ^9^-tetrahydrocannabinol (THC) and low levels of other phytocannabinoids, particularly cannabidiol (CBD). Use of this type of cannabis is thought by some to predispose to greater adverse outcomes on mental health and fewer therapeutic benefits. Australia has one of the highest *per capita* rates of cannabis use in the world yet there has been no previous systematic analysis of the cannabis being used. In the present study we examined the cannabinoid content of 206 cannabis samples that had been confiscated by police from recreational users holding 15 g of cannabis or less, under the New South Wales “Cannabis Cautioning” scheme. A further 26 “Known Provenance” samples were analysed that had been seized by police from larger indoor or outdoor cultivation sites rather than from street level users. An HPLC method was used to determine the content of 9 cannabinoids: THC, CBD, cannabigerol (CBG), and their plant-based carboxylic acid precursors THC-A, CBD-A and CBG-A, as well as cannabichromene (CBC), cannabinol (CBN) and tetrahydrocannabivarin (THC-V). The “Cannabis Cautioning” samples showed high mean THC content (THC+THC-A = 14.88%) and low mean CBD content (CBD+CBD-A = 0.14%). A modest level of CBG was detected (CBG+CBG-A = 1.18%) and very low levels of CBC, CBN and THC-V (<0.1%). “Known Provenance” samples showed no significant differences in THC content between those seized from indoor versus outdoor cultivation sites. The present analysis echoes trends reported in other countries towards the use of high potency cannabis with very low CBD content. The implications for public health outcomes and harm reduction strategies are discussed.

## Introduction

Analysis of the cannabinoid content of cannabis plants is of interest given the likelihood that both the medicinal effects and adverse health effects of cannabis consumption may be dictated by the concentration and interplay of certain phytocannabinoids. There is international concern over research findings suggesting that contemporary cannabis cultivation is biased towards plants with high levels of Δ9-tetrahydrocannabinol (THC), the cannabinoid responsible for most of the psychoactive effects of cannabis, and negligible levels of cannabidiol (CBD), and other trace cannabinoids, that have therapeutic potential and may counteract some of the unpleasant effects of THC [1]. A general theme of these concerns is whether cannabis is somehow a “different” drug to that consumed in previous decades, and whether increased THC content and/or diminished levels of CBD and other trace cannabinoids is accentuating adverse effects of cannabis on mental health.

Research over the past few decades in the United Kingdom, Europe, the United States and New Zealand, has identified an increase in the concentration of THC in herbal cannabis [2], [3], [4], [5], [6], [7]. For example, US data indicate that herbal cannabis contained an average of 3.4% THC and 0.3% CBD in 1993, while in 2008 THC levels more than doubled to 8.8% with CBD remaining low (0.4%) [5]. There is, however, evidence of a stabilisation in THC content in the UK and parts of Europe since peaks in the late 1990s/early 2000s [3], [8]. There also remains considerable variability in THC levels within and across studies, as well as according to location, season, quality and freshness and type of cannabis (e.g., very high levels in Dutch *niederweet*; *sinsemilla* vs. ditchweed vs. *hashish*) [2], [5], [6], [7], [9], [10], [11]. Despite these caveats, more recent short-term studies of cannabis seizures in disparate geographic regions confirm a consistent pattern of a predominance of THC and low or negligible levels of other important cannabinoids such as CBD, particularly in samples identified as *sinsemilla* [12], [13], [14]. While there have been sporadic early reports of individual samples containing high THC levels [15], it has been proposed that this current pattern may be linked to a number of factors, including selective breeding of certain cannabis strains with a high THC/low CBD level, a preference for female plants (*sinsemilla*), the rise of widespread intensive indoor cannabis cultivation (a controlled growing environment), and global availability of seeds and equipment over the internet [6], [7], [8], [9].

A high THC/low CBD cannabinoid profile has been linked to a number of putative outcomes, including increased risks for cannabis dependence [16], and increases in treatment seeking for cannabis-related problems [8], although there is little research systematically addressing the public health impacts of use of different strengths and types of cannabis. There is suggestive evidence from analyses of cannabinoids in hair samples that regular users with a high THC/low CBD profile in hair may have increased vulnerability to psychosis relative to users with a more balanced THC/CBD profile [17], [18], [19]. This is consistent with laboratory research showing that CBD may prevent or inhibit the psychotogenic and memory-impairing effects of THC [20], [21], [22]. While the evidence for the ameliorating effects of CBD is not universal [1], [18], [23] it is thought that consumption of high THC/low CBD cannabis may predispose users towards adverse psychiatric effects, relative to the use of cannabis with more moderate THC/higher CBD content.

Recent major policy responses in several countries have reflected these concerns. For example, in justifying their decision in 2008 to reclassify cannabis as a category Class B drug after previously downgrading it to a Class C drug in 2004, the UK Home Office stated: “The significant increase in both the market share of higher than average potency cannabis and its actual potency in the last few years in the UK are compelling factors” [24]. More recently in the Netherlands, the Garretsen Commission recommended that cannabis with a THC level of greater than 15% be classified as a “hard drug” due to the high THC levels in contemporary Dutch cannabis which “increased the risks for public health” [25].

Globally, Australia has one of the highest rates of cannabis use [26], [27], while the occurrence of population indicators of cannabis-related harm, including hospital separations for cannabis-induced psychosis and cannabis-related problems such as dependence, increased over the 2000s [28]. 2010–11 cannabis detections at the Australian border, the majority of which were seeds with total weight less than 1 gram, were the highest on record, while the scale of the domestic market means that importation of herbal cannabis is negligible [29]. During this same period the number of national cannabis seizures and arrests were the highest on record [29]. Despite this, there is no legal imperative to test for cannabis potency, and thus no formal testing program. This study, therefore, provides the first comprehensive Australian data on street-level cannabis potency, through analysis of cannabis seizures obtained from New South Wales (NSW), Australia’s most populous state. An additional aim was to compare whether there were differences in the profiles of outdoor-grown and indoor-grown cannabis. In addition to examining levels of THC, we analysed levels of cannabinoids that have therapeutic potential, and which might antagonise or synergise certain THC effects (including CBD).

## Materials and Methods

### Sample Acquisition

Two separate groups of cannabis seizures were analysed, comprising:

- cannabis seizures confiscated by NSW Police between October 9, 2010 and October 19, 2011, as part of the Cannabis Cautioning Scheme. Under this scheme, adults detected by police using or in possession of not more than 15 g of dried cannabis and/or equipment for using the cannabis may receive a formal police caution rather than face criminal charges and court proceedings. As these seizures are not required for evidentiary purposes but destroyed by police, permission was received from NSW Police to analyse them for this study. The origin and cultivation method of these samples was therefore unknown. Samples were obtained from 23 police commands in NSW, with 39.8% of samples from rural/regional areas and 60.2% from urban/metropolitan areas. The rural/regional areas sampled were located in parts of NSW long associated with the “counter-culture”, and have entrenched associations with cannabis use and cultivation, particularly using outdoor methods. Only seizures containing at least 2 g of green plant material (GPM) were eligible for analysis; those containing tobacco were rejected. Of the 200 seizures obtained in sealed exhibit bags, 195 (97.5%) contained one piece of GPM, 4 (2%) contained two pieces of GPM (2%) and 1 (0.5%) contained 3 pieces of GPM, resulting in a total of n = 206 samples for analysis. These are referred to as “Cannabis Cautioning” samples.
- GPM obtained during NSW police cannabis crop eradication operations between February and May, 2012. Samples were collected from thirteen different outdoor soil-grown cannabis crops (size from a dozen to 500 plants) raided during police operations against commercial growing interests on the rural mid-northern NSW coast, a prominent cannabis cultivation area. The thirteen indoor soil-grown crops (size of 100 to 300 plants) were obtained during police operations in urban Sydney. Together these indoor and outdoor larger scale seizures are referred to as “Known Provenance” samples.

### Sample Storage

Storage and analysis of all samples was undertaken in a secure laboratory in the Discipline of Pharmacology, University of Sydney. On receipt, samples were photographed and weighed and stored at −20°C in a locked freezer.

### Sample Preparation

As Cannabis Cautioning samples were not uniform in form and appearance, plant material used for analysis was selected from the female buds of cannabis samples to minimise variation due to sampling bias. The extraction procedure used was based on a validated protocol [30]. Samples were then dried for 24 h in a 35°C forced ventilation oven. Dried samples were crumbed, ground and mixed. 200 mg of this fine powder were weighed in a glass vial and extracted with 10 mL of a mixture of methanol/chloroform (v/v: 9/1) by sonication for 30 min. The extract was filtered and appropriately diluted in an amber vial. A 100 µL aliquot of the dilution was evaporated under a nitrogen stream and redissolved in 100 µL of a mixture of water/acetonitrile (v/v: 5/5) containing diazepam (50 mg/L) as an internal standard. Two separate extractions were performed on each sample, and these were separately assayed and compared.

### Chromatographic Analysis

Analysis of cannabinoid content was undertaken using high performance liquid chromatography diode array detection (HPLC-DAD) using the method of De Backer *et al.* [30] with slight modification. The modified method was validated (for selectivity, linearity, accuracy, precision and recovery) according to the currently accepted USA Food and Drug Administration (FDA) guidance for bioanalytical method validation [31]. The calibration range was linear from 2 µg/ml to 100 µg/ml, and cannabinoid concentrations greater than 100 µg/ml were diluted to ensure the reading within the calibration range. Quality control samples (3 different cannabinoid mixture levels) were incorporated into each HPLC run to ensure the validity of the data collected. Accuracy (average bias = 4.2%) and precision (average coefficient of variation (CV) = 3.8%) were all within acceptable confidence limits. Recovery efficiency was further validated from re-extracted powder samples.

The following cannabinoids were analysed: Δ9-tetrahydrocannabinol (THC), cannabidiol (CBD), cannabigerol (CBG), cannabichromene (CBC), cannabinol (CBN) and tetrahydrocannabivarin (THC-V); in addition, the carboxylic acid precursor molecular forms of Δ9-tetrahydrocannabinol (THC-A), cannabidiol (CBD-A) and cannabigerol (CBG-A), which are more plentiful in raw plant material, were also quantified.

The HPLC system consisted of a Shimadzu ADVP module (Kyoto, Japan) equipped with a SIL-10 autoinjector with sample cooler and LC-10 in-line vacuum degassing solvent delivery unit. Chromatographic separation of all cannabinoids and internal standard (IS) diazepam was accomplished on a Waters X-Bridge C18 (4.6 mm×150 mm, 3.5 micron) reverse-phase column (Waters, Australia) coupled with a 1 mm Opti-Guard C18 pre-column (Optimize Technologies, Alpha Resources, Thornleigh, Australia) maintained at 25°C by a Shimadzu CTO-10AS column oven (Kyoto, Japan).

**Figure 1:**
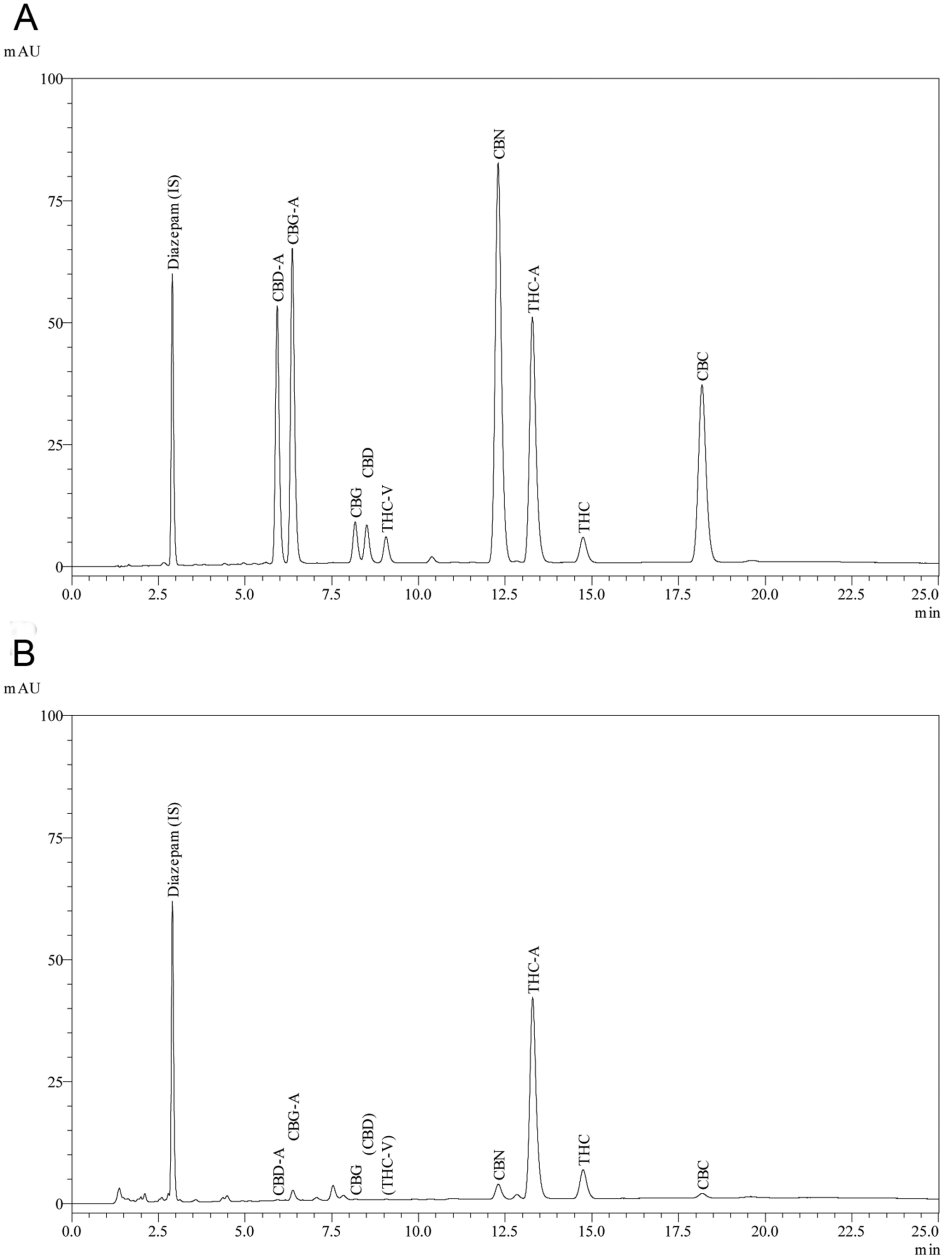
Chromatograms of analysed cannabinoids. A) Chromatogram of calibration standard mixture of all analysed cannabinoids at 100 µg/ml. B) Representative chromatogram of a typical “Cannabis Cautioning” seized sample.

The linear gradient solutions consisted of mobile phase (A) 50 mM ammonium formate buffer pH 3.75 with 10% acetonitrile, and (B) 90% acetronitrile, with the following elution program utilised, 0 min, 70% B; 15 min, 90% B; 30 min, 90% B; 31 min, 70% B and 40 min 70%. The flow rate was maintained at 1 ml/min. The eluate from the column was monitored at 272 nm via SPD-M20A diode array detector (Kyoto, Japan). The injection volume of reconstituted extract was 5 µl. Chromatographic control, data collection and processing were carried out using Shimadzu Class VP data software (version 7.4, Kyoto, Japan). Quantitation of unknown concentrations of cannabinoids and control samples were obtained from the linear regression equation of calibration curves of individual reference standards by plotting concentration versus the area ratio of the standard and internal standard. Control and representative chromatograms are shown in Figure 1.

All analyses were conducted with two separate extracts of each individual sample. Individual cannabinoid values are expressed as w/w %. In addition to the 9 cannabinoid values quantified (above), we also calculated the total content of THC (THC_tot_), CBD (CBD_tot_) and CBG (CBG_tot_), using formulae which adjusted for the differing molecular weight of the cannabinoid and carboxylic conjugative components of each cannabinoid [32]:
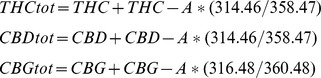



### Statistical Analysis

There were strong positive correlations between the major cannabinoid values in the duplicate extracts from samples used in HPLC analyses (Cautioning samples: THC_tot_ r^2^ = 0.81, CBD_tot_ r^2^ = 0.61, CBG_tot_ r^2^ = 0.79; Known Provenance samples: THC_tot_ r^2^ = 0.68, CBD_tot_ r^2^ = 0.41, CBG_tot_ r^2^ = 0.96). Thus, for each cannabinoid, the mean values obtained from the two runs were used in statistical analyses.

The majority of distributions for cannabinoid content were skewed. For those that were normally distributed, we checked for outliers using the method of Mehmedic and colleagues [5]; no outliers were detected and thus no values were excluded from analysis. Descriptive statistics (w/w %: mean, median and range) are presented for each cannabinoid analysed for both the Cannabis Cautioning and Known Provenance samples. Differences in cannabinoid content between urban and rural seizure locations (in the Cannabis Cautioning samples) and between indoor- and outdoor-grown seizures (in the Known Provenance samples) were analysed using t-tests for normally distributed variables and the non-parametric Median test for skewed distributions. Each of these sets of analyses was adjusted for multiple testing using Bonferroni adjustments (α = 0.05/12 = 0.004 required; α = 0.01/12 = 0.0008 etc.) to control for Type 1 error. All analyses were conducted using IBM SPSS Statistics Version 20 or Prism GraphPad 6.0.

## Results

### Cannabinoid Profiles in Cannabis Cautioning Samples

**Figure 2:**
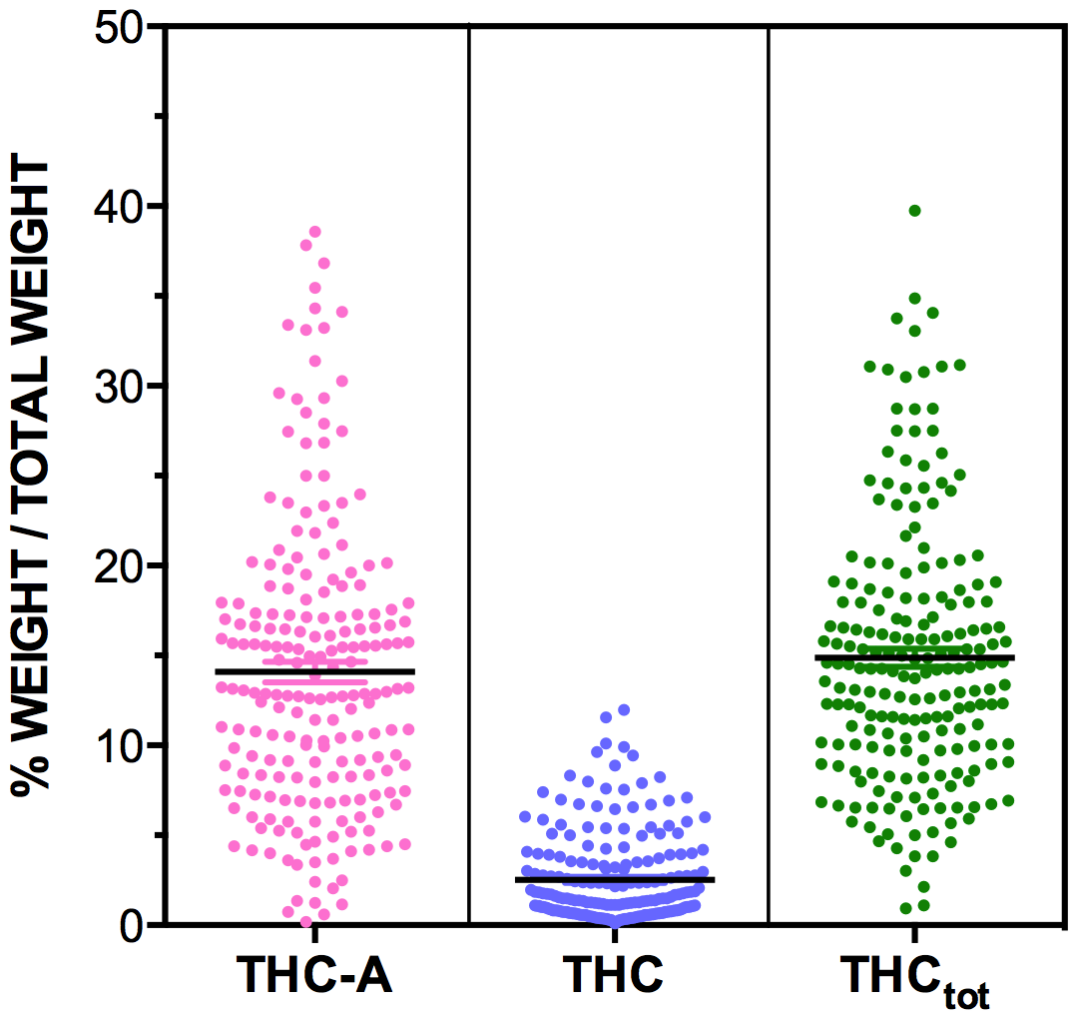
The levels of THC-A, THC and THC_tot_ measured in n = 206 Cannabis Cautioning seizures from NSW. Levels of cannabinoids are expressed as % of total weight of sample (w/w%). THC_tot_ levels are obtained from adding the amount of free THC seen in the cannabis to the amount found in the non-psychoactive from of THC-A while adjusting for the differing molecular weight of the cannabinoid and carboxylic conjugative components of each cannabinoid (THC_tot_ = THC+THC-A*(314.46/358.47)).

**Figure 3:**
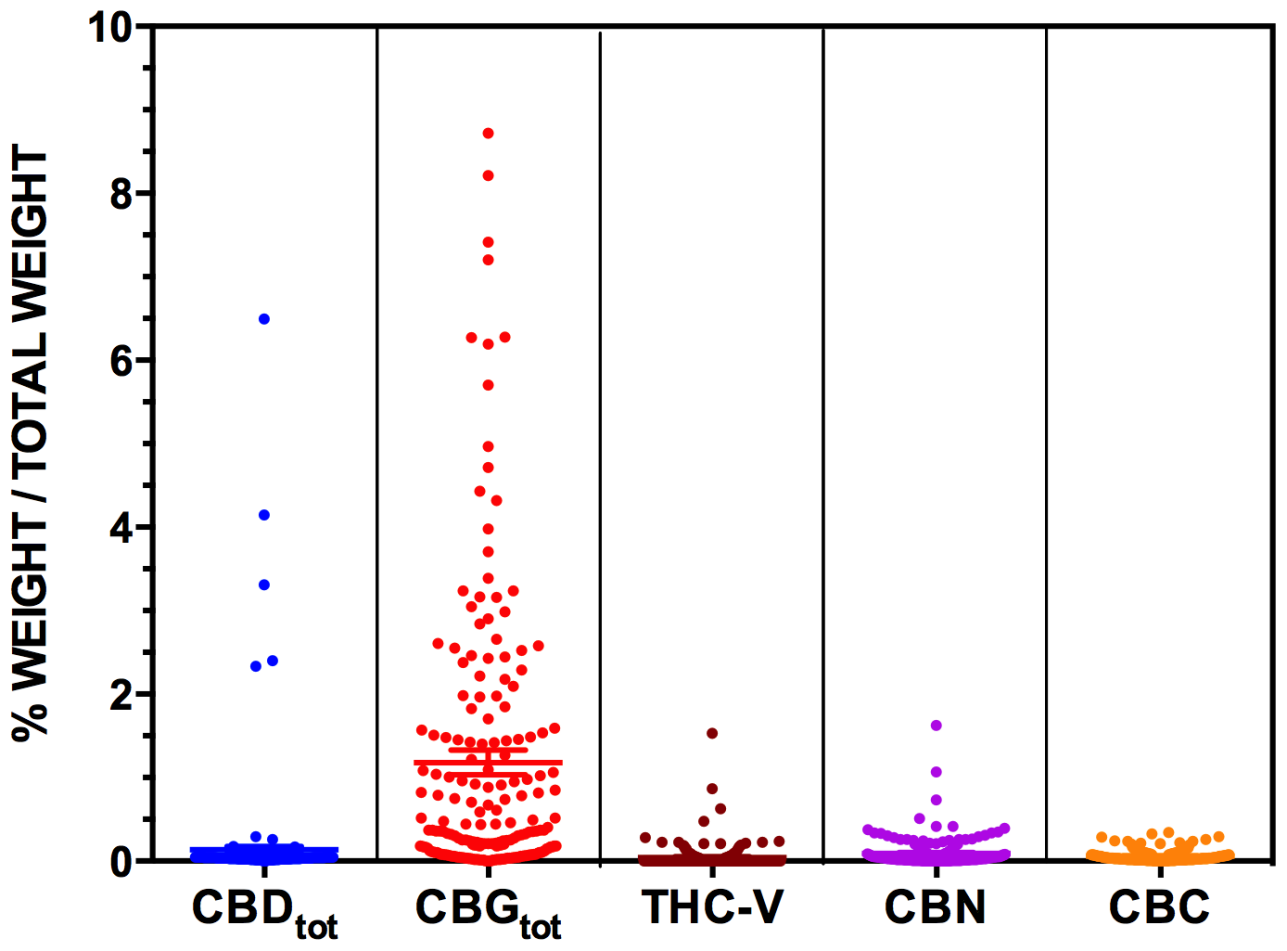
The levels of CBD_tot,_ CBG_tot_, THC-V, CBN and CBC measured in n = 206 Cannabis Cautioning seizures from NSW. Levels of cannabinoids are expressed as % of total weight of sample (w/w%). Note the differing scales relative to Figure 2. Note that the CBG_tot_ levels of two samples are not shown on the graph as they are out of scale (values = 15.83% and 13.77%).

The results from the Cannabis Cautioning samples are presented in Table 1. As shown in Figures 2 and 3 the cannabinoid content of these samples was dominated by THC and THC-A, with low levels of all other cannabinoids analysed. As expected, levels of THC-A were far greater than THC, showing the dominance of the carboxylic acid precursor in plant materials. Absolute levels of THC_tot_ in the Cannabis Cautioning samples ranged from 0.9% to 39.8% (Figure 2).

**Table 1:** Profile of cannabinoid content (w/w%) in n = 206 samples of cannabis seized during the NSW cannabis cautioning program (October 2010–October 2011).

	Range	Mean (95% CI)	Median	Mean (%) USA 2008^1^		Median UK 2005^2^	
				marijuana	sinsemilla	herbal	sinsemilla
**THC-A**	0.18–38.59	14.08 (12.97–15.20)	12.95				
**THC**	0.08–11.98	2.52 (2.18–2.86)	1.45				
**THC_tot_**	0.94–39.76	14.88 (13.87–15.88)	14.26	5.83	11.5	2.1	13.98
**CBD-A**	0–4.34	0.10 (0.04–0.16)	0.04				
**CBD**	0–2.69	0.04 (0.01–0.08)	0				
**CBD_tot_**	0–6.50	0.14 (0.05–0.22)	0.04	0.4	0.2	<0.1	<0.1
**CBG-A**	0–2.61	0.28 (0.22–0.34)	0.13				
**CBG**	0–14.98	0.93 (0.66–1.20)	0.08				
**CBG_tot_**	0–15.83	1.18 (0.89–1.47)	0.32	0.3	0.4	0.2	0.4
**CBN**	0–1.62	0.09 (0.07–0.11)	0.03	0.3	0.2	0.6	0.2
**CBC**	0–0.34	0.06 (0.05–0.07)	0.03	0.2	0.3	0.2	0.2
**THC-V**	0–1.53	0.04 (0.02–0.06)	0	0.1	0.1	0.2	<0.03

1 Mehmedic et al, *J Forensic Sci, 55*, 1209–1217, 2011. Marijuana comprises leaves, stems, seeds and flowering tops; sinsemilla defined as flowering tops of unfertilised female plants with no seeds (n = 46,211).

2 Potter et al, *J Forensic Sci, 53*, 90–94, 2008. Herbal cannabis defined as imported cannabis, characteristics as for marijuana (above); sinsemilla defined as above (n = 452).

3 For all mean/median USA and UK values of THC, CBD and CBG, it is not known if reported values represent total of THC-A and THC, or simply THC.

### Cannabinoid Profiles in Known Provenance Samples

**Figure 4:**
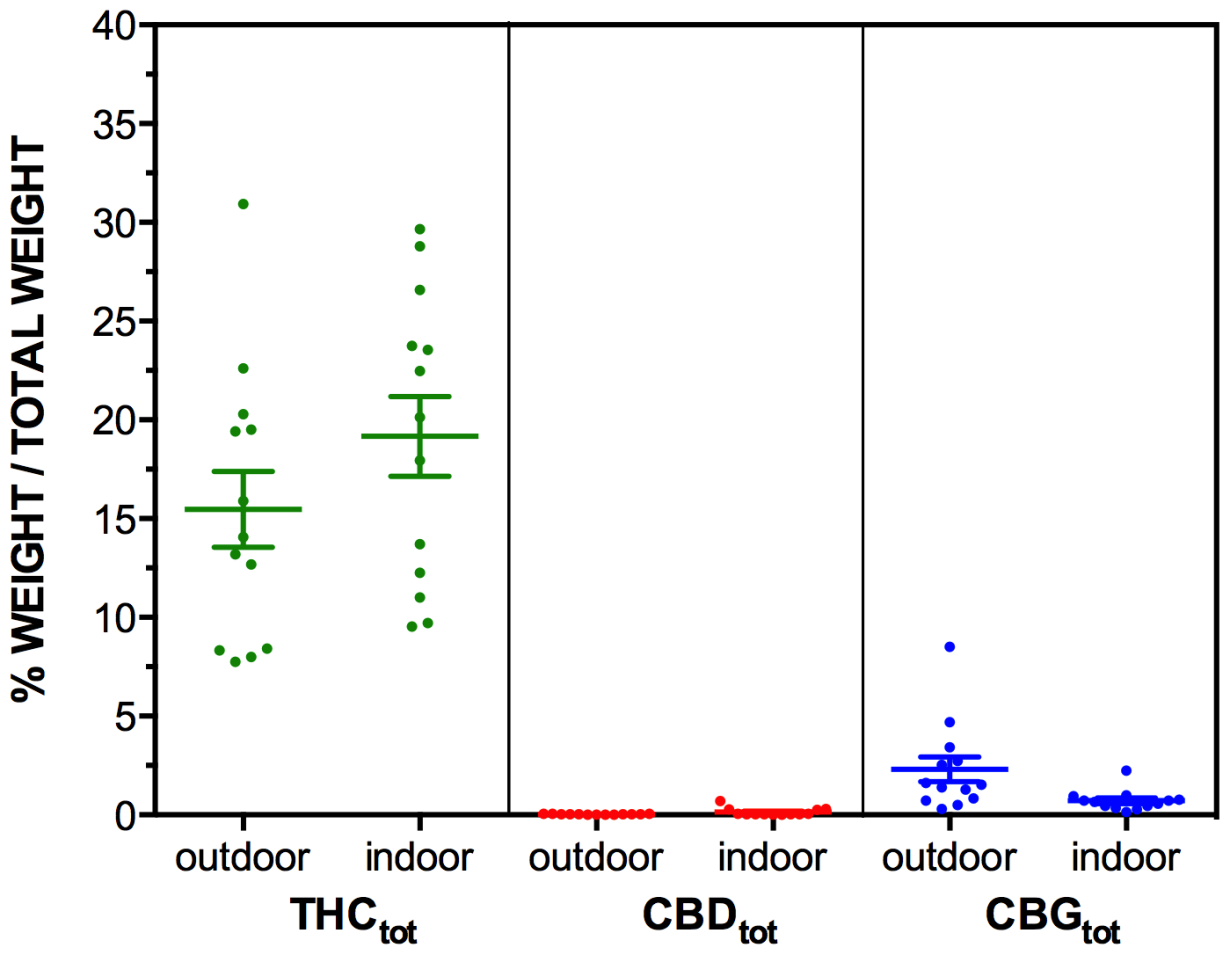
The levels of THC_tot_, CBD_tot_ and CBG_tot_, in n = 13 outdoor grown (NSW North Coast) and n = 13 indoor grown (Sydney) “Known Provenance” samples. Levels of cannabinoids are expressed as mean % of total weight of sample (w/w%), with ± SEM bars shown.

**Figure 5:**
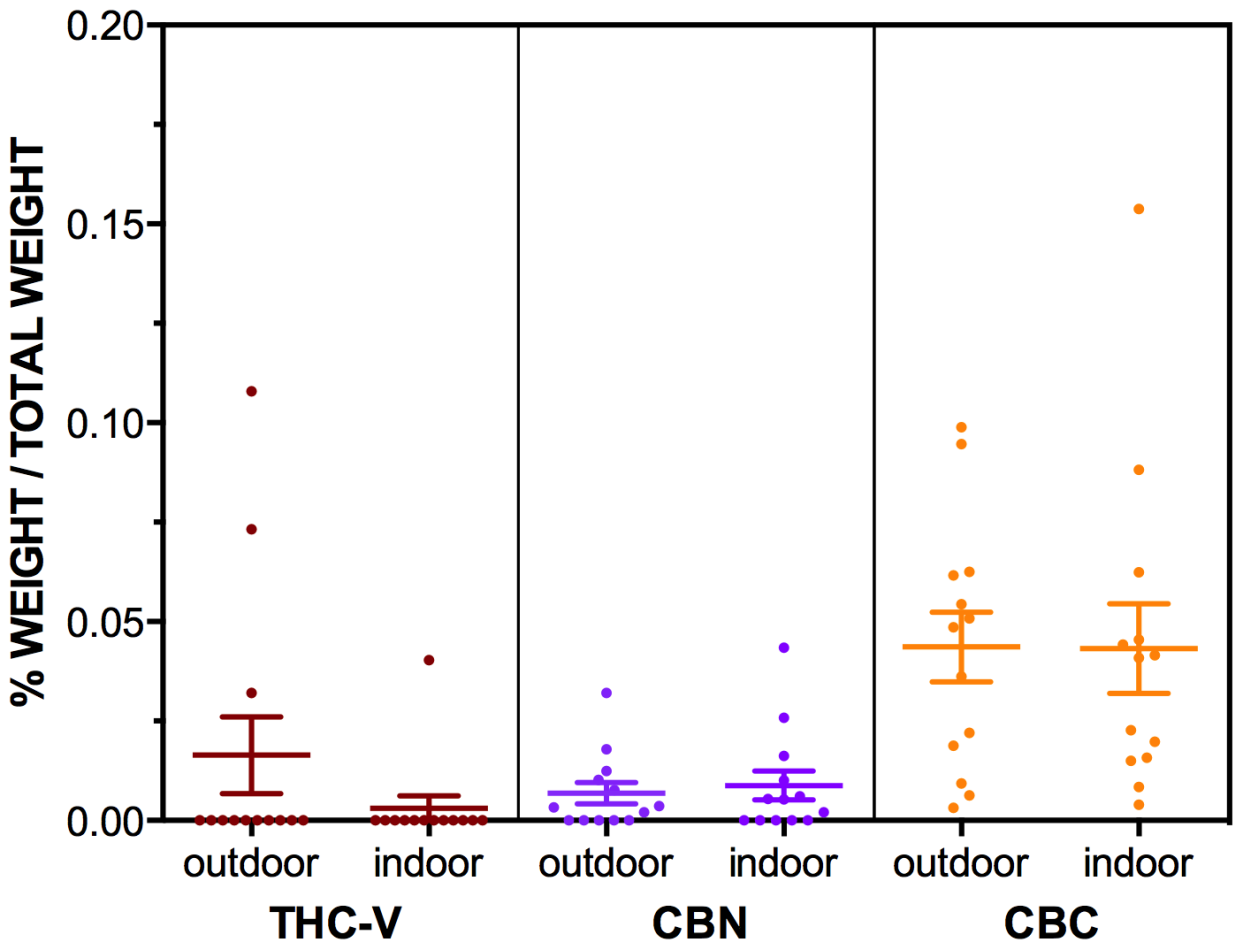
The levels of THC-V, CBN and CBC in n = 13 outdoor grown (NSW North Coast) and n = 13 indoor grown (Sydney) “Known Provenance” samples. Levels of cannabinoids are expressed as mean % of total weight of sample (w/w%), with ± SEM bars shown. Note the differing scales relative to Figure 4.

The results from the Known Provenance samples are presented Table 2 and in Figures 4 and 5. Results were broadly consistent with the Cannabis Cautioning samples with high levels of THC-A and THC, and low levels of all other cannabinoids in samples from both indoor and outdoor locations.

**Table 2:** Profile of cannabinoid content (w/w%) in Indoor and Outdoor Grown “Known Provenance” cannabis samples seized during the NSW cannabis eradication program, February-May 2012.

	Indoor Grown (n = 13)			Outdoor Grown (n = 13)		
	Range	Mean (95% CI)	Median	Range	Mean (95%CI)	Median
**THC-A**	9.58–33.12	19.57 (14.55–24.58)	17.52	8.15–34.24	16.46 (11.73–21.20)	14.68
**THC**	0.18–8.18	2.00 (0.54–3.47)	1.10	0.15–2.77	1.03 (0.60–1.45)	0.90
**THC-tot**	9.54–29.66	19.16 (14.76–23.57)	20.13	7.75–30.93	15.47 (11.28–19.66)	14.07
**CBD-A**	0.02–0.06	0.04 (0.03–0.04)	0.04	0.01–0.08	0.04 (0.02–0.05)	0.04
**CBD**	0–0.67	0.11 (0–0.23)	0	0	0 (-)	0
**CBD-tot**	0.02–0.70	0.14 (0.02–0.26)	0.04	0.01–0.07	0.03 (0.02–0.04)	0.04
**CBG-A**	0.03–0.92	0.29 (0.15–0.44)	0.30	0.12–2.37	0.67 (0.25–1.09)	0.43
**CBG**	0–2.16	0.46 (0.12–0.79)	0.33	0.18–6.43	1.73 (0.65–2.80)	1.09
**CBG-tot**	0.14–2.24	0.71 (0.40–1.03)	0.65	0.29–8.51	2.32 (0.96–3.67)	1.53
**CBN**	0–0.04	0.01 (0–0.02)	0.01	0–0.03	0.01 (0–0.01)	0
**CBC**	0–0.15	0.04 (0.02–0.07)	0.04	0–0.10	0.04 (0.02–0.06)	0.05
**THC-V**	0–0.04	0 (0–0.01)	0	0–0.11	0.02 (0–0.04)	0

Despite a wide range, 74% of street-level Cannabis Cautioning samples and 77% of Known Provenance samples contained at least 10% THC_tot_. Further, 43% of Cannabis Cautioning and 54% of Known Provenance samples contained at least 15% THC_tot_, the level recommended by the Garretsen Commission as warranting classification of cannabis as a “hard” drug in the Netherlands.

The samples contained comparatively high amounts of CBG_tot_, with 1.18% in the Cannabis Cautioning samples and 2.32% CBG_tot_ and 0.71% CBG_tot_ in the outdoor and indoor Known Provenance samples. A total of 31% of all Cannabis Cautioning samples and 38% of all Known Provenance samples contained at least 1% CBG_tot_.

Conversely, levels of all other cannabinoids with potential therapeutic value were negligible, and comprised only a fraction of the content of samples compared to THC and THC-A (Figures 2 and 3). Notably, 91% of Cannabis Cautioning samples and 85% of Known Provenance samples contained less than 0.1% CBD_tot_.

### Differences in Urban/Rural Cannabinoid Levels

Among the Cannabis Cautioning Samples, samples seized from rural locations differed in cannabinoid content from those seized from urban locations. Rural samples showed higher levels of THC (median 3.55% vs. 0.94%; p<0.0001), THC-A (mean 16.48% vs. 12.50%; p = 0.0005) and THC_tot_ (mean 18.66% vs 12.38%, p<0.0001). Rural samples also contained higher levels of CBD_tot_ (median 0.05 vs. 0.03; p<0.0001), CBG (median 1.43 vs. 0, p<0.0.0001), CBG-A (median 0.22 vs 0.10; p = 0.0006), CBG_tot_ (median 1.58 vs. 0.17, p<0.0.0001), CBN (median 0.09 vs. 0.02, p<0.0.0001), CBC (median 0.09 vs. 0.02, p<0.0.0001) and THC-V (median 0.03 vs. 0, p<0.0001).

However, without knowledge of the sources of these samples, it is not possible to identify whether urban and rural seizures are likely to represent cannabis grown using different cultivation methods. That is, it is possible that Cannabis Cautioning samples obtained in rural seizures had been grown in urban locations, and vice versa. To address this issue, samples of known origin were also tested (Figure 4 and 5), with indoor samples sourced from Sydney, and outdoor samples seized from the North Coast area of NSW.

### Differences Indoor/Outdoor Cannabinoid Levels

Results showed no differences in cannabinoid levels between Known Provenance seizures from indoor or outdoor grown crops, although there was much cross-over in distributions, and there was a trend towards higher THC_tot_ values in indoor grown seizures.

## Discussion

These analyses confirm global trends towards the dominance of THC content in contemporary cannabis, with these Australian data showing average values similar, if not slightly higher, than recent international studies (Table 1). While there was wide variation in cannabinoid levels, high mean and median values of THC_tot_ and low values of CBD_tot_ and other potentially therapeutic cannabinoids are similar to those reported internationally in samples of cannabis identified as *sinsemilla*, commonly referred to as “skunk” [3], [5], [7].

This pattern of high THC/low CBD cannabis has become a focus of concerns over the potential mental health impacts of current cannabis use patterns. Given existing data on the potential modulating effects of CBD on the adverse effects of THC, these data lend support to the proposition that cannabis currently available in Australia exhibits a profile that may render some cannabis users vulnerable to potential adverse mental health impacts of their use. However, there remains scant research on this issue other than small scale surveys and laboratory studies demonstrating biological plausibility. For example, while there have been noted increases in treatment seeking for cannabis use internationally across the past decade, particularly in young people, there are other conceivable explanations apart from increased potency. These might include improved treatment availability and schemes where users are diverted from the criminal justice system into treatment [33]. Further, while Australian hospital separations for cannabis-induced psychosis increased over the 2000s, particularly among older age groups [28], modelling research does not indicate increases in levels of schizophrenia commensurate with increases in cannabis use [34], [35].

There are also several possible moderators of the impacts of cannabis potency on cannabis users. While there is mixed evidence on use trends, overall cannabis use appears to be stabilising or declining in some regions (e.g., Western Europe, USA and Australia) after increased use throughout the 1990s and early 2000s [8], [26]. Further, *effective* potency, that is the amount of THC and other relevant cannabinoids actually absorbed by the user, may vary according to such factors as natural variations in the cannabinoid content of plants, the part of the plant consumed (e.g., more potent buds versus leaf material), route of administration (e.g., oral vs. smoking) and user titration of dose to compensate for differing levels of THC in different smoked material [10], [36]. In smoking cannabis, only approximately 30% of THC-A is thought to be converted to free THC [37] with THC, rather than THC-A, providing the main psychoactive effects when cannabis is smoked or vaporized. Thus, THC_tot_ may not necessarily be an accurate representation of *effective* potency. On the other hand, the non-psychoactive THC-A content of plants is of increasing interest given its potential medicinal and neuroprotective properties [38]. A recent trend towards “juicing” cannabis plant material for consumption is aimed at maximising THC-A intake, while minimising the intoxicating effects of THC.

Although the overall median levels of CBD were very low in our samples it is interesting to note there were 5 samples that exceeded 1% CBD_tot_, with one containing 6.5% CBD_tot_. Of these 5 high CBD_tot_ samples identified, 4 were seized from rural locations and 1 seized from an urban location. CBD-A is also gathering attention for its therapeutic potential, with evidence of anti-emetic [39] and anti-cancer properties [40]. Samples obtained from rural seizures contained higher levels of virtually all measured cannabinoids including trace phytocannabinoids, but most noticeably THC, THC-A and THC_tot_. This is not entirely surprising given that regional areas of NSW such as Byron Bay, Lismore and Tweed Heads have long been associated with cannabis use and specialist cultivation approaches. However, it is not currently possible to identify whether urban and rural Cannabis Cautioning seizures are likely to represent cannabis grown using different cultivation methods as the origin of the samples was unknown, and could even reflect cannabis grown hydroponically in urban locations and transported to regional NSW.

There were no differences between known outdoor (Northern NSW) and indoor (Sydney) grown seizures in levels of THC, CBD or other cannabinoids, although there were trends towards higher THC_tot_ in indoor-grown samples. These data therefore do not provide overwhelming support for claims of higher potency in cannabis grown using intensive indoor cultivation techniques. Given the observed trends towards higher THC, this small sample may have had insufficient power to reliably detect such differences. However, it may simply be that specific types of seed material are favoured for cannabis cultivation, and that this factor dominates cannabinoid profiles rather than the use of outdoor or indoor growing locations. An interesting issue for future research is the value growers place on strains containing high levels of THC and low levels of CBD, as reflected in their preference among many cultivators [8], [41] and higher market prices [6], [8], [42]. Given concerns over the potential mental health impacts of this profile, as well as reports of the aversive nature of the high associated with it by some users [43], research on user preferences associated with different effects might shed light on whether cannabis containing a more balanced mix of THC and CBD would have value in the market, as well as potentially conferring reduced risks to mental wellbeing.

There were relatively high levels of CBG_tot_ (the precursor molecule to THC-A, CBD-A and CBC-A [32]) when compared to other trace phytocannabinoids, with CBG the second most abundant phytocannabinoid in the seized plant material. Research has found that CBG-A increases up to the twelfth week of cultivation (third week of flowering) and then decreases until the end of cultivation, while CBG increases all the way to the end of cultivation [44]. High CBG in seized cannabis plants may indicate that growers may be allowing their plants to mature before harvesting. As a weak partial agonist at cannabinoid type1 (CB1) and type 2 (CB2) receptors, a highly potent α_2_ adrenoceptor agonist, and a moderately potent serotonin-1A (5HT_1A_) antagonist [45], there may be a potential use for CBG as an antidepressant and analgesic [46].

We also found trace amounts of the non-psychotropic phytocannabinoid THC-V, which appears to have an antagonistic effect on CB1 receptors, displacing synthetic CB1 agonists CP-55940 and WIN-55212 and attenuating the antinociceptive and hypothermic effects of THC *in vivo* [47]. However, the THC-V concentrations used to produce an antagonistic response are at least 100–1000 times higher than what would be reasonably absorbed during smoking of a typical joint. CBC, another trace non-psychotropic phytocannabinoid appears to modulate the effect of THC by inhibiting endocannabinoid cellular reuptake, and is also a potent activator of TRPA1 receptors, with apparent analgesic [48] and anti-inflammatory effects [49], [50]. However, like CBD, the trend for maximising THC production may have led to marginalisation of CBC as historically, CBC has sometimes been reported to be the second or third most abundant cannabinoid [51].

Some limitations inherent in the data presented here should be acknowledged. Due to funding constraints we could not collect a very large random or necessarily representative sample of Cannabis Cautioning seizures. However, we did ensure the samples we obtained came from the major rural cannabis growing areas on the NSW north coast and the major urban areas of the state. Further, as both Cannabis Cautioning and Known Provenance samples were not required to be retained for criminal proceedings, we received and stored them soon after they were seized. The freshness of the samples is confirmed by the dominance of carboxylic acid forms of THC, CBD and CBG, and very low levels of CBN, the main oxidation product of THC.

Given the known variability of THC within a single plant [3], it is possible that these results do not represent the “true” average potency of each plant as buds were used whenever possible from samples that were analyzed. However, there were strong positive correlations between the duplicate analyses for the samples. While these data are cross-sectional, the profile we reported is nevertheless highly consistent with that of international samples. Routine longitudinal monitoring, the analysis of larger samples of cannabis grown using known cultivation methods, and sampling from multiple parts of the plant would assist us in better understanding potency trends and the impacts of cultivation technique on cannabinoid profile.
